# Implications of genomic signatures in the differential vulnerability to fetal alcohol exposure in C57BL/6 and DBA/2 mice

**DOI:** 10.3389/fgene.2014.00173

**Published:** 2014-06-11

**Authors:** Amy C. Lossie, William M. Muir, Chiao-Ling Lo, Floyd Timm, Yunlong Liu, Whitney Gray, Feng C. Zhou

**Affiliations:** ^1^Department of Animal Sciences, Purdue UniversityWest Lafayette, IN, USA; ^2^Department of Medicine, Indiana University School of MedicineIndianapolis, IN, USA; ^3^Department of Anatomy and Cell Biology, Indiana University School of MedicineIndianapolis, IN, USA; ^4^Department of Molecular and Medical Genetics, Indiana University School of MedicineIndianapolis, IN, USA; ^5^Stark Neuroscience Research Institute, Indiana University School of MedicineIndianapolis, IN, USA

**Keywords:** fetal alcohol syndrome, gene x environment interactions, genomics, gene expression, next generation sequencing, genetic association, epigenetics

## Abstract

Maternal alcohol consumption inflicts a multitude of phenotypic consequences that range from undetectable changes to severe dysmorphology. Using tightly controlled murine studies that deliver precise amounts of alcohol at discrete developmental stages, our group and other labs demonstrated in prior studies that the C57BL/6 and DBA/2 inbred mouse strains display differential susceptibility to the teratogenic effects of alcohol. Since the phenotypic diversity extends beyond the amount, dosage and timing of alcohol exposure, it is likely that an individual's genetic background contributes to the phenotypic spectrum. To identify the genomic signatures associated with these observed differences in alcohol-induced dysmorphology, we conducted a microarray-based transcriptome study that also interrogated the genomic signatures between these two lines based on genetic background and alcohol exposure. This approach is called a gene x environment (GxE) analysis; one example of a GxE interaction would be a gene whose expression level increases in C57BL/6, but decreases in DBA/2 embryos, following alcohol exposure. We identified 35 candidate genes exhibiting GxE interactions. To identify *cis*-acting factors that mediated these interactions, we interrogated the proximal promoters of these 35 candidates and found 241 single nucleotide variants (SNVs) in 16 promoters. Further investigation indicated that 186 SNVs (15 promoters) are predicted to alter transcription factor binding. In addition, 62 SNVs created, removed or altered the placement of a CpG dinucleotide in 13 of the proximal promoters, 53 of which overlapped putative transcription factor binding sites. These 53 SNVs are also our top candidates for future studies aimed at examining the effects of alcohol on epigenetic gene regulation.

## Introduction

Women who drink during pregnancy place their unborn children at risk of acquiring clinical features of fetal alcohol syndrome (FAS) or fetal alcohol spectrum disorder (FASD). FASD encompasses all patients displaying some of the clinical features of fetal alcohol exposure and is much more prevalent than FAS. These syndromes comprise an array of phenotypes that include: cognitive deficits, intrauterine and post-natal growth retardation, memory deficits, poor motor skills, facial dysmorphology and social/behavioral problems. On one end of the spectrum (mild FASD; ~1 in 100 live births), children present with mild to moderate mental deficits that are difficult to diagnose by appearance, while those on the other end (FAS; ~1 in 1000 live births) exhibit a readily identifiable facial dysmorphology coupled with a complement of severe neuropsychological sequelae and neurobehavioral deficits (Abel, 1995; Stratton et al., 1996; Sampson et al., 1997; Jacobson, 1998; Astley et al., 2002; Niwa et al., 2002; Hoyme et al., 2005; Moore et al., 2007).

Several factors contribute to the phenotypic variability observed within FAS/FASD, including quantity, frequency and duration of alcohol exposure (Coles, 1993; Abel, 1995; Maier and West, 2001; May et al., 2013), developmental stage at the time of consumption and underlying maternal factors and/or genetic background influences, as only 5–10% of women with a positive drinking history give birth to children displaying features of FASD (Abel, 1995; Stratton et al., 1996). Furthermore, monozygotic twins display phenotypes that are more similar than dizygotic twins, suggesting that genetic background affects the incidence of FAS/FASD (Christoffel and Salafsky, 1975; Chasnoff, 1985; Streissguth and Dehaene, 1993; Riikonen, 1994). In addition, allelic variation within the alcohol dehydrogenase gene, *ADH1B*, can lead to varying teratogenic outcomes in different ethnic groups (Warren and Li, 2005). These reports support the hypothesis that environmental elements, plus an individual's genetic background, contribute significantly to his or her susceptibility to the teratogenic effects of alcohol.

There are challenges to studying the pathogenesis of FAS/FASD in the human population; the clinical drinking history is not always complete or reliable, and it is unethical to conduct fetal alcohol studies in humans. To address these challenges, several groups performed studies describing the morphological changes that occur following alcohol exposure in various strains of mice. Two inbred mouse strains, C57BL/6 and DBA/2, differ widely in their response to maternal consumption of alcohol (Gilliam et al., 1988; Goodlett et al., 1989; Ogawa et al., 2005; Downing et al., 2009, 2012; Chen et al., 2011; Zhou et al., 2011). Previous studies by our group demonstrated that while DBA/2 animals are resistant to the teratogenic effects of fetal alcohol exposure in embryonic cultures, C57BL/6 mice are quite susceptible, exhibiting phenotypic abnormalities that affect embryonic lethality, brain morphology, fetal weight gain, behavior, as well as formation of the digits, skeleton, eyes, kidneys and heart (Ogawa et al., 2005; Chen et al., 2011; Zhou et al., 2011). Using a whole embryo culture approach that strictly controls the timing and dose of alcohol exposure, without interference from the maternal environment, we demonstrated that genetic background was a driving factor in the teratogenicity of alcohol in C57BL/6 embryos (Ogawa et al., 2005; Chen et al., 2011).

In this report, we sought to better understand the underlying genomic vulnerabilities to the teratogenic effects of alcohol observed between C57BL/6 and DBA/2 cultured embryos. We hypothesized that by strictly controlling confounding physiological factors (i.e., the maternal environment, intrauterine position effects, etc.), these studies would identify underlying genetic differences between C57BL/6 and DBA/2 that contribute to the observed differential vulnerability to the teratogenic effects of alcohol. Furthermore, we proposed that polymorphisms between C57BL/6 and DBA/2 in regulatory elements and other factors that influence RNA expression are responsible for much of these morphological differences. We used a two-pronged approach that takes advantage of the strain-specific genomic signatures and gene expression profiles to identify candidate *cis*-regulatory elements with the potential to drive differential expression between C57BL/6 and DBA/2 embryonic cultures. First, we used a gene x environment (GxE) approach to identify transcripts that were affected by both genetic background (i.e., C57BL/6 vs. DBA/2) and alcohol exposure (presence vs. absence of alcohol) to identify genes that are candidates for the morphological diversity between the these two inbred strains following exposure of equimolar ratios of alcohol in cultured embryos. Since the GxE approach identifies differentially expressed genes that are candidates for mediating the interaction, we then sought to uncover underlying single nucleotide variants (SNVs) in *cis*-regulatory elements within the proximal promoters of these candidate genes. SNVs within the promoters are excellent candidates for regulating expression of the nearby altered genes, as regulatory SNVs can alter the location and function of enhancers and promoters (De Gobbi et al., 2006) that change transcriptional levels (Munkhtulga et al., 2010) in specific cell types (Poitras et al., 2010) and lead to allele-specific changes in expression (Azad et al., 2013). SNV studies narrowed the list to 16 genes that showed both a GxE interaction based on gene expression studies and an SNV driven to fixation in opposite directions in the proximal promoter. Pathway analysis of this set of genes enhanced the understanding of the pathophysiology of alcohol exposure. Lastly, we investigated how these SNVs may lead to altered DNA methylation and transcription factor binding, with 53 SNVs potentially affecting both DNA methylation and transcription factor binding. This study provides a framework for future experiments aimed at understanding the combinatorial effects of these SNVs on susceptibility to FAS/FASD, and these two strains provide a tractable model system for identifying the complement of genetic loci and epigenetic events that contribute to the teratogenesis that occur along the spectrum of FAS/FASD.

## Materials and methods

### Mouse strains used in this study

The Harlan strains used in the microarray study (C57BL/6NHsd and DBA/2NHsd), as well as the lines used to identify the potential regulatory SNVs (C57BL/6NJ and DBA/2J), descended from the original C57BL/6J (http://jaxmice.jax.org/strain/000664.html) and DBA/2J (http://jaxmice.jax.org/strain/000671.html) lines created at The Jackson Laboratories (Zurita et al., 2011). The C57BL/6 founder line originated from one of Abbie Lathram's fancy mouse stocks in 1921 by CC Little, and was maintained by brother-sister mating at the Jackson Laboratories until 1951 (at generation F32), when a sub-line, designated C57BL/6N, was sent to the NIH (Zurita et al., 2011). This sub-line is a founder line for C57BL/6NHsd and C57BL/6NJ. Harlan acquired these animals from the NIH in 1983 and has maintained them continuously in their colony; this sub-line is designated C57BL/6NHsd (order code 044; http://www.harlan.com). The C57BL/6NJ (http://jaxmice.jax.org/strain/005304.html) sub-line consists of embryos that were frozen at the NIH in 1997 and sent back to the Jackson Laboratory in 2005. The Jackson Laboratory thawed this line, expanded it and froze it down immediately. New stock animals are continually thawed to maintain the genetic integrity of the C57BL/6NJ sub-line. CC Little started inbreeding DBA/2 mice in 1909, and DBA/2J animals have been maintained continuously at The Jackson Laboratories. A sub-line (DBA/2N) was sent to the NIH in 1951 (at generation F34). Harlan later acquired this line, designated DBA/2NHsd (order code 042; http://www.harlan.com).

### Alcohol exposure in embryonic cultures

All experimental procedures were approved by the Institutional Animal Care and Use Committee of the Indiana University School of Medicine (Indianapolis, IN) and are in accordance with the guidelines of the Institutional Animal Care and Use Committee of the National Institute on Drug Abuse, National Institutes of Health, and the Guide for the Care and Use of Laboratory Animals (National-Academy-of-Sciences, 2010).

Previous studies from our lab measured the phenotypic variations between the C57BL/6 and DBA/2 inbred strains resulting from alcohol exposure in embryonic cultures (Ogawa et al., 2005; Chen et al., 2011). The technique for whole embryo culture was described previously (Ogawa et al., 2005), based on methods by New (New, 1978). Briefly, 2-month-old C57BL/6NHsd and DBA/2NHsd mice from Harlan, Inc. (Indianapolis, IN) were individually housed and acclimated for at least 1 week prior to mating. Two females were placed with one male of the same strain for 2 h. When a vaginal plug was detected after the mating period, it was designated as embryonic day 0 (E0). On E8.25, females were sacrificed by CO_2_ asphyxiation for morphological and transcriptome studies.

The alcohol exposure paradigm that yielded the highest degree of differential vulnerability was used for gene expression analysis (Chen et al., 2011). The gravid uterus was removed at 37°C; each embryo, plus the visceral yolk sac and a small piece of the ectoplacental cone, was carefully removed. Three embryos from each C57BL/6NHsd or DBA/2NHsd dam, bearing 3–5 somites (~E8.25, age of the embryos is determined by the somites), were collected. Four groups of embryos, C57BL/6NHsd with or without alcohol and DBA/2NHsd with or without alcohol (*n* = 6 each) were incubated in 20 mL of medium, consisting of 70% immediately centrifuged heat-inactivated rat serum and 30% phosphate buffered saline supplemented with 20 units/ml penicillin and 20 units/ml streptomycin (Sigma, St. Louis, MO). Culture bottles were gassed for 2 h with 5% O_2_, 5% CO_2_ and 90% N_2_ in a rotating culture system (B.T.C. Precision Incubator Unit, B.T.C. engineering, Cambridge, England, 36 rpm). After a 1–2 h acclimation period, a 6 h treatment was initiated by incubating embryos in fresh medium with or without 88 mM ethanol in isotonic buffer. After the 6-h alcohol treatment, all embryos were transferred to alcohol-free culture media for 15–16 h, for a total of 24 h in culture. The 6 h exposure allows a precise delineation of the developmental time period in which various teratogenic effects can be induced, which facilitates a rigorous examination of the temporal windows of vulnerability (Chen et al., 2011).

In previous studies, we demonstrated distinct morphological differences between these two lines following 44 h of continuous ethanol treatment (Ogawa et al., 2005) and 42 h after a 6 h ethanol treatment (Chen et al., 2011). In both studies, the C57BL/6 embryonic cultures were more susceptible to the teratogenic effects of alcohol than the DBA/2 animals. However, the two lines showed some differential dysmorphology after alcohol exposure. For example, C57BL/6 animals showed increased developmental delay compared to DBA/2 animals, although both are delayed compared to controls (Chen et al., 2011). In the same study, we cataloged the embryonic structures that showed morphological evidence of developmental delay in C57BL/6 and DBA/2 cultures. For example, dysmorphology is prominent in the heart, forebrain, midbrain, hindbrain, caudal neural tube, optic vesicle and hindlimb in C57BL/6 cultures, but restricted to the forebrain and optic vesicle in DBA/2 cultures. Furthermore, C57BL/6 embyros contained a large number of cleaved(c)-caspase 3 positive (+) cells (i.e., apoptotic cells) in the optic vesicles, several brain regions, the craniofacial primordial, as well as cranial nerve nuclei V, VII, VIII, and IX following alcohol exposure. In contrast, alcohol-exposed DBA/2 embryos only contained a small number of c-caspase 3^+^ cells (Chen et al., 2011).

In the present study, our goal was to capture the transcriptome changes that occur prior to development of the morphological changes in order to gain an understanding of the genetic pathways that are targeted by alcohol exposure, not necessarily the alterations in expression that occur after manifestation of the dysmorphology. We hypothesized that collecting embryos at 15–16 h post-alcohol treatment would give the culture system time to eliminate the alcohol and reduce potential alcohol-induced artifacts. Therefore, all cultures were terminated 24 h after the start of alcohol treatment to reveal the upstream genetic pathways. Embryos were alive at termination, which was confirmed by observing a beating heart and circulating blood in the yolk sac. We conducted morphological examinations of the embryos at embryonic day (E) 8.25 (when culture initiated), +1 day in culture (i.e., E8.25 + 1); E8.25 + 1 is analogous to an embryo examined at E9.0. We observed several morphological changes in the embryos, which are analogous to those observed at the later time points (Ogawa et al., 2005; Chen et al., 2011). In all cases, the C57BL/6NHsd embryos demonstrated more morphological changes and developmental delay compared to the DBA/2NHsd strain. In addition, we noticed two distinct subtypes of embryos in C57BL/6NHsd cultures. Approximately 50% of the ethanol-treated C57BL/6NHsd cultures still had an open neural tube. This could be due to delayed embryonic development or to the teratological effects of alcohol. Therefore, we stratified the embryonic cultures into two subtypes, neural tube closed (NTC) and neural tube open (NTO) in an attempt to capture distinct genetic differences potentially caused by alcohol. All control embryos from both genotypes (C57BL/6NHsd and DBA/2NHsd) had a closed neural tube. Following alcohol treatment, we observed that the majority of the DBA/2NHsd embryos also had a closed neural tube. However, the neural tube failed to close in a small number of DBA/2NHsd and ~50% of the C57BL/6NHsd embryos, resulting in an NTO morphology. Therefore, we subclassified each embryo into NTO and NTC bins. Embryos were segregated into six subtypes based on neural tube dysmorphology (NTO vs. NTC), inbred strain (C57BL/6NHsd or DBA/2NHsd) and alcohol exposure [with EtOH vs. without (Ctrl)]: NTO-B6-EtOH, NTC- B6-EtOH, NTC-B6-Ctrl, NTO-D2-EtOH, NTC-D2-EtOH and NTC-D2-Ctrl, where B6 indicates C57BL/6NHsd and D2 designates DBA/2NHsd. For more strict and detailed comparison, we analyzed the NTC and NTO embryos separately compared to their respective NTC control embryos. Due to the small numbers of NTO embryos in the DBA/2NHsd background, a large number of DBA/2NHsd animals were used in this study.

### Affymetrix gene expression

The comparative expression studies presented here were conducted on heads instead of whole embryos to focus on the cranial neural crest and brain. At this stage (~E9.0), further dissection of parts of the head or brain would not yield enough tissue for microarray studies. The head of each embryo was dissected above the otic vesicle, immediately immersed in 0.7 ml TRIzol (Invitrogen, Carlsbad, CA) and homogenized to extract total RNA for RT-PCR and microarray studies. Total RNA was isolated from 36 individual embryos (6 vehicle control, 6 NTC alcohol treated and 6 NTO alcohol treated per strain) as previously described (Zhou et al., 2011). The quality of RNA was assessed by the Agilent Bioanalyzer (Agilent Technologies, Waldbronn, Germany) and by spectrophotometry (220 nm to 350 nm); concentration was determined from the values at A260. Microarray hybridizations and bioinformatics analyses were performed at the Center for Medical Genomics at the Indiana University School of Medicine. Labeling and hybridization to Affymetrix Mouse Genome 430A GeneChips® (Affymetrix, Santa Clara, CA) were carried out following the manufacturer's suggested procedure. The Mouse Genome 430A chip contains over 22,600 probe sets representing transcripts and variants from over 14,000 well-characterized mouse genes. Fragmented biotinylated RNA from each embryo was hybridized to its own GeneChip for 17 h at 42°C. We previously demonstrated that the microarray analysis revealed striking differences between the embryos with open neural tubes (EtOH-NTO) and those with closed neural tubes (EtOH-NTC) (Zhou et al., 2011). Control embryos had closed neural tubes. Therefore, the transcriptome analysis was conducted based on the embryonic morphological phenotype.

The data from independent arrays (each with RNA from a single embryo) for each treatment were extracted using the Affymetrix Microarray Suite 5.0 (MAS5) algorithm. Data for both experiments have been deposited in GEO/NCBI and have been assigned series accession number GSE9545 and sample numbers GSM241642 through GSM241660. To minimize false positive results, only genes detected (“present” by the MAS5 algorithm) on at least half of all individual arrays in at least one experimental condition were retained for further analysis. This avoids data that primarily represent noise (McClintick and Edenberg, 2006).

### B6 and D2 genomic sequence

We remapped the recently established genomic sequence available at the European Nucleotide Archive (http://www.ebi.ac.uk/ena/) for both C57BL/6NJ (http://www.ebi.ac.uk/ena/data/view/ERS076384&display=html) and DBA/2J (http://www.ebi.ac.uk/ena/data/view/ERS075663&display=html) onto release 66 of the *M. musculus* genome from Ensemble (http://www.ensembl.org) using CLC Genomics Workbench V4.7 (http://www.clcbio.com). We then called SNVs using the quality based variant detection tool with the neighborhood radius set to 5, maximum gap and mismatch count set to 2, minimum neighborhood quality of PHRED 15, and minimum central quality of PHRED 20. Variants in non-specific regions were ignored. Across all reads, a minimum variant frequency greater than 10% was required.

### Gene X environment (GxE) interactions

The transcriptome of these lines, as impacted by alcohol exposure, was quantified by microarray analysis. We designed this experiment to analyze expression based on embryo neural tube dysmorphology (NTO and NTC), genetic background (C57BL/6NHsd or DBA/2NHsd) and alcohol exposure (88 mm EtOH or vehicle) in six groups as indicated in the experimental design above. Five embryos from each category were used for microarray studies. Since there were no control animals with the neural tube open morphology, all NTO studies used the control-NTC animals for comparison.

We analyzed the data using the Affymetrix Expression Console 1.1.2800.19935. Intensities were normalized using quantile normalization and the adjusted data were analyzed using the probe logarithmic intensity error (PLIER) method. According to the Affymetrix manual (http://media.affymetrix.com/support/technical/technotes/plier_technote.pdf). *The PLIER method produces a more accurate probe set signal by employing feature responses to interpret intensity data, dynamic weighting by empirical feature performance, and handling error appropriately across low and high target abundance.* The results from the PLIER analysis were variance stabilized using log_2_ transformation and analyzed by gene for significant differences using a 2 factor analysis of variance adjusted for multiple testing with a false discover rate of 5% (Benjamini and Hochberg, 1995). The main effects were line (DBA/2J vs. C57BL/6NHsd) and exposure to alcohol. Of primary interest were those genes where a differential response to alcohol was dependent on the line, i.e., differential expression in response to alcohol is dependent on the genetic background.

### Identifying candidate SNVs

Since the C57BL/6NHsd and DBA/2NHsd genomes are not available, we chose closely related sub-strains (C57BL/6NJ and DBA/2J) for the genomic analyses. To define potential SNVs driving the differential phenotype following alcohol exposure, we cataloged all SNVs between C57BL/6NJ and DBA/2J located within 5 kb upstream of the transcriptional start site(s) of each candidate gene. To define a SNV, we calculated the difference in allele frequency for the same allele between C57BL/6NJ and DBA/2J, and concentrated on alleles driven to opposite fixation. SNVs were defined as alleles that were fixed at ≥0.90 (i.e., the frequency was ≥90% in one strain and ≤10% in the other). To identify putative transcription factor binding sites, we retrieved 20 bp of flanking sequence (10 bp on each side of the SNV; 21 bp total) from the NCBI37/mm9 mouse genome assembly, and put the sequence into PROMO (http://alggen.lsi.upc.es/) (Messeguer et al., 2002; Farre et al., 2003). PROMO uses version 8.3 of Transfac (Knuppel et al., 1994; Wingender, 2008) to identify putative transcription factor binding sites. For genes expressed from the Watson strand, we used the published sequence; for genes expressed on the Crick strand, we used the reverse complement. This ensured that we analyzed the same strand that the transcriptional machinery uses to transcribe the gene. The C57BL/6NJ and DBA/2J sequences were analyzed separately for putative transcription factor binding sites and additions or loss of CpG dinucleotides.

## Results

### Line-specific differences in gene expression

**Figure 1:**
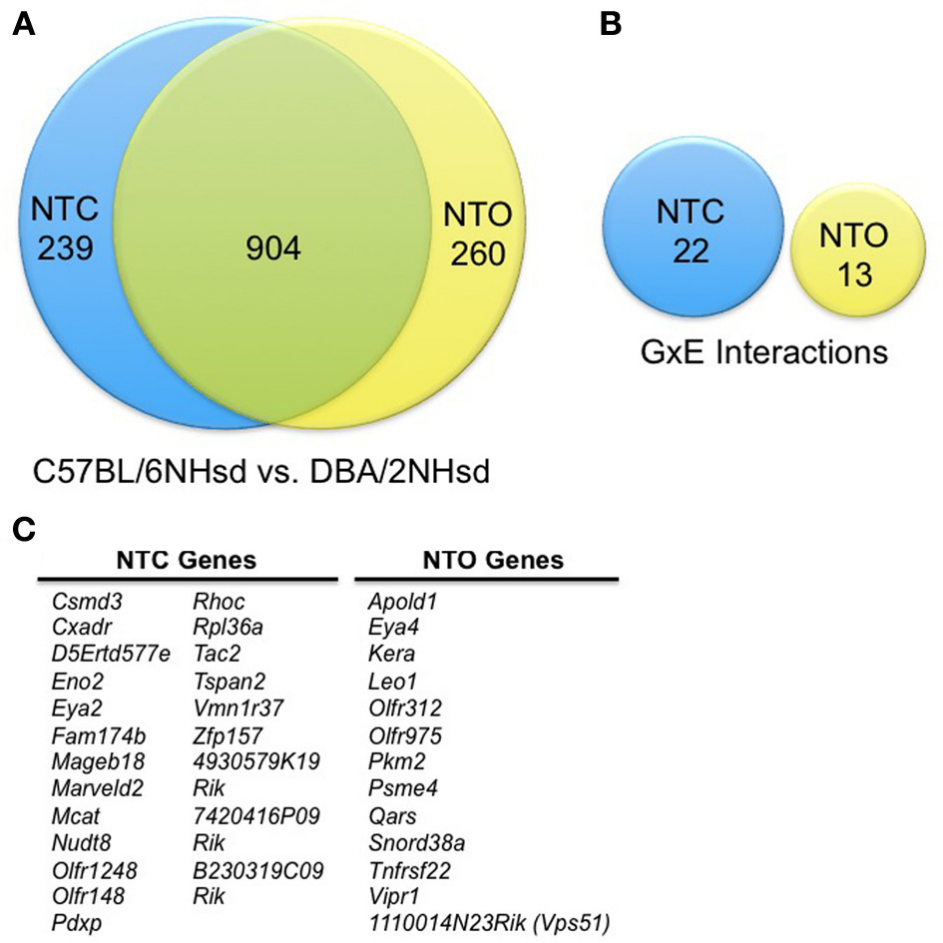
**Summary of the differentially expressed genes**. **(A)** Line-specific differences in gene expression. We conducted this analysis in two separate experiments that captured two embryonic phenotypes: neural tube closed (NTC) and neural tube open (NTO). This analysis was conducted by examining the gene expression changes solely between C57BL/6NHsd and DBA/2NHsd; alcohol exposure is not taken into consideration. Therefore, in the NTC group, all C57BL/6NHsd embryos (with and without alcohol) were measured as one cohort, while the DBA/2NHsd embryos (with and without alcohol) comprised the second group. The NTO cohort was analyzed in the same manner. This depicts the innate changes in gene expression between the two genetic backgrounds, plus the changes that are due to alcohol exposure, but cannot assess if there are interactions between the genetic background and alcohol exposure. There are 1403 genes that are differentially expressed between the C57BL/6NHsd and DBA/2NHsd embryos; 904 of these are differentially expressed in both the NTO and NTC studies. **(B)** Differential gene expression as result of genetic and alcohol interactions (GxE). As above, we analyzed gene expression in the NTC and NTO cohorts separately. In this study, we measured the potential interactions between alcohol exposure and genetic background. We found 22 genes that demonstrated GxE interactions in the NTC group and 13 genes showing GxE interactions in the NTO group. **(C)** Genes subject to GxE Interactions. The genes subject to GxE interactions are delineated.

We first compared gene expression profiles between all C57BL/6NHsd and all DBA/2NHsd cultured embryos without taking alcohol exposure into consideration. We combined all C57BL/6NHsd embryos with an NTC morphology, which included those exposed and not exposed to alcohol, and compared them to the DBA/2NHsd embryos with a NTC phenotype, including those exposed and not exposed to alcohol. We repeated this study with embryos displaying NTO. However, in this analysis, the controls had a NTC phenotype, as none of the control animals displayed an NTO morphology. This analysis was used to determine the subset of genes that are differentially expressed between the two lines. Using a false discovery rate (FDR) of 5% (Benjamini and Hochberg, 1995), we identified 1143 genes that were differentially expressed in NTC embryos and 1164 genes that were differentially expressed in NTO embryos, for a total of 1403 differentially expressed genes between these two inbred strains (Figure 1A). In these two independent experiments, 904 differentially expressed transcripts were detected in both the NTC and NTO embryos; ~718 are significant at an FDR of ~10^−5^. To confirm that these demonstrate statistical significance, we performed a hypergeometric distribution and a binomial distribution. Using a hypergeometic distribution, we found that the probability of drawing 904 genes that would be in the union by chance with independent draws is less than 0.015. A more informative metric might be the number one would expect by chance. This can be approximated with the binomial distribution because the numbers are large, i.e., sampling with replacement gives probabilities similar to sampling without replacement. The proportion of genes found significant in each experiment was 0.0327 and 0.0324 respectively. With independence, the proportion expected in the union is 2(0.0327) (0.0324) × 35,561 = 75. We observed about 12 times that number.

Many genes in this cohort are key to the development of the nervous system including: channel proteins and receptors, e.g., *Kcnmb1* (potassium large conductance calcium-activated channel), *Chrnb1* [cholinergic receptor, nicotinic, beta polypeptide 1 (muscle)]; neural transcription factors and homeobox genes, e.g., *Nkx1-2* [NK1 transcription factor related, locus 2 (Drosophila)], *Igf1* (insulin-like growth factor 1), *Gbx2* (gastrulation brain homeobox 2), *Sox21* (SRY (sex determining region Y)-box 21); epigenetic components, e.g., *Hist1h2ab* (histone cluster 1, H2ab), *Setd7* (SET domain containing lysine methyltransferase 7); heat shock proteins, e.g., *Dnajc13, Dnajc21* DnaJ (Hsp40) homolog, subfamily C, member 13 and 21; genes involved in apoptosis, e.g., *Bnip3l* (BCL2/adenovirus E1B interacting protein 3-like), *Gadd45gip1* (growth arrest and DNA-damage-inducible, gamma interacting protein 1), *E2f5* (E2F transcription factor 5); and structural proteins, *Bean1* (brain expressed, associated with Nedd4, 1), *Tuba3a* (tubulin, alpha 3A), *Optc* (opticin).

Furthermore, Ingenuity Pathway Analysis (IPA; Ingenuity® Systems, www.ingenuity.com) of this set of 904 genes found in both the NTC and NTO experiments indicates that the most significant pathways are: inflammatory response; cell death and survival; energy production; lipid metabolism; embryonic development; and cancer, hematological disease. We picked three networks as examples. The genes delineating these networks are listed in Table 1.

**Table 1:** **Networks of NTO/NTC overlapping genes between C57BL/6NHsd and DBA/2NHsd**.

**Molecules in network**	**Score**	**# Target genes**	**Top diseases and functions**
*Ager, Atp5f1, Atp5g2, B3gnt2, Bcl2l14, Ccl19, Ccl21, Ccl27, Clcn7, Cyba, Epx, Fgf18, Gfi1, Gipc1, Gp49a/Lilrb4, Gzmk, Hmgb2, Itgb1bp1, Metap2, Mgat3, Mkl2, Nod1, Ntsr2, Oscp1, Ppp2r2d, Prg3, S100a1, S100a9, Smg1, Sytl1, Tff2, Tpm4*	50	32	Inflammatory Response, Cellular Function and Maintenance, Cellular Movement
*Akt2, Arhgap23, Atf6, Birc6, Camkk1, Chkb, Cox7a2l, Dcps, Ddx24, Dennd4a, Gapdh, Hmgb1, Hpcal1, Kdelr1, Map3k5, Myef2, Pank2, Prex1, Smek2, Smndc1, Stxbp4, Syncrip, Tinf2, Tomm22, Vdac3, Vps28, Ywhaq, Znrf2*	40	28	Cellular Compromise, Cellular Function and Maintenance, Cell Death and Survival
*Actg2, Anapc1, Blvra, Cdc23, Cml3, Cpd, Cxxc4, Daz2, Dlx5, Dvl1, Ercc4, Gde1, Hbg2, Hmbs, Hsd3b4, Kirrel, Let-7, Mir-204, Runx2, Sbf1, Sfrp4, Smad4, Sprr1a, Tob2, Trim32*	34	25	Embryonic Development, Organ Development, Organismal Development

Notably among the 1403 differentially expressed genes, a number of microRNAs were identified: *miR206, miR31, miR463, miR551b, miR669a-2, miR142, miR211, miR22, miR27b, miR326, miR429, miR488, miR672, miRlet7a-2, miRlet7d, miRlet7e, miRlet7i, miR330, miR683-1*. Many of these miRNAs display functions relevant to development. For example *miR-142* regulates the formation and differentiation of hematopoietic stem cells in vertebrates (Lu et al., 2013). *miR-31*, which plays multiple roles during development and in cancer progression, is over expressed in myoblasts from patients with Duchene Muscular Dystrophy, compared to control individuals (Cacchiarelli et al., 2011) and thought to act as an inhibitor of metastasis in breast cancer cells (Valastyan et al., 2011). While *miR-206* regulates angiogenesis in zebrafish (Stahlhut et al., 2012) the miRlet7 family is essential for cell fate determination in C. elegans (Reinhart et al., 2000) and has been implicated in impairment of tumorigenesis (Johnson et al., 2005; Kumar et al., 2008). Finally, *miR-27b* regulates CYP1B1 expression post-transcriptionally in cancer tissues (Tsuchiya et al., 2006; Chuturgoon et al., 2014).

### Differential gene expression as result of genetic and alcohol interactions (GxE)

To further understand the genes that are responsive to alcohol and display a genetic basis, we determined which transcripts showed an interaction between these two conditions (i.e., a gene x environment (GxE) interaction). We compared the ethanol-treated animals with a NTC subtype from each genotype to their respective NTC controls. However, since none of the control animals had an NTO phenotype, NTC embryos from the same strain were used as a control, as follows: the Control-NTC vs. Alcohol-NTC group and the Control-NTO vs. Alcohol-NTO group. Accepting an FDR of 5%, we identified 35 candidates that demonstrate a GxE interaction; 22 were in the NTC subtype and 13 were in the NTO subtype (Figures 1B,C). IPA studies indicate that the top networks are cancer; respiratory disease; cell-to-cell signaling; gene expression; cell death and survival; and cellular compromise (Table 2). These 35 genes are top candidates underlying the divergent morphological changes observed between C57BL/6Hsd and DBA/2Hsd embryonic cultures that were exposed to alcohol.

**Table 2:** **Genes Demonstrating a GxE Interaction with Associated SNVs in the Promoter**.

**Gene**	**Name**	**Function**	**Expression during mouse development**	**Relevance to neural development**	**References**
*Eya2*	*Eyes Absent Homolog2 (Drosophila)*	Tyrosine phosphatase, transcription activator	Cranial placodes, branchial arches and CNS during organogenesis	Regulate preplacedal ectoderm (PPE) differentiation	Xu et al., 1997; Grifone et al., 2007; Sato et al., 2010
*Csmd3*	*CUB and Sushi multiple domains 3*	Hydrolyzing O-glycosyl compounds	E14.5 to adult—Cardiovascular and nervous system (mainly in human adult and fetal brains)	Candidate gene for Autism Spectrum Disorders (ASDs)	Shimizu et al., 2003; Floris et al., 2008
*Mcat*	*Malonyl CoA:ACP Acyltransferase (Mitochondrial)*	Transfer a malonyl group from malonyl-CoA to the mitochondrial acyl carrier protein	E14.5 by RT-PCR	N/A	Smith et al., 2012
*Tac2*	*Tachykinin-2*	Neural peptide signaling	E11.5 to adult—CNS	May involve pain modulation	Mar et al., 2012
*Vps51*	*Vacuolar protein sorting 51*	Transport of endosomes to the trans-Golgi network (TGN)	N/A	N/A	Skarnes et al., 2011
*Apold1*	*Apolipoprotein L Domain Containing 1*	Angiogenesis and activity-dependent changes of brain vasculature	E14.5—Heart ventricle	May affect blood-brain permeability	Diez-Roux et al., 2011; Zhou et al., 2013
*Leo1*	*Paf1/RNA Polymerase II Complex Component*	Gene transcription regulation and chromatin remodeling	E9.5, E11.5, and E14.5—urogenital system	Cardiac and neural crest development in zebrafish	Wertz and Herrmann, 2000
*Psme4*	*Proteasome (Prosome, Macropain) Activator Subunit 4*	Proteasome activator, involves in histone degradation and DNA damage response	E14.5—Nervous system, sensory organs, alimentary system, and skeletal system	N/A	Ustrell et al., 2002; Qian et al., 2013
*Vipr1*	*Vasoactive Intestinal Peptide Receptor 1*	VIP receptor, modulated by G-proteins	E12-E18.5 by RT-PCR; detected at	Promote neuronal survival	Delgado et al., 1996; Fabricius et al., 2011
			E13.5—cerebral cortex; Adult rat: cerebral cortex and hippocampus		
*Fam174b*	*Family with Sequence Similarity 174, Member B*	Membrane protein of unknown function	E14.5—alimentary system, limbs, head, skeleton system, sensory organs	Deleted in a patient with behavioral disturbance, autism, and epilepsy.	Diez-Roux et al., 2011; Sarahan et al., 2011; Kamien et al., 2014
*Mageb18*	*Melanoma-associated antigen B18*	Infection, inflammation, regulation of cell proliferation and apoptosis	Post-natal brain, testis and adipose tissue	N/A	Diez-Roux et al., 2011; Lin et al., 2012
*Pdxp*	*Pyridoxal (pyridoxine, vitamin B6) phosphatase*	Vitamin B6 Metabolism	E14.5—Nervous system, sensory organs, cardiovascular system, alimentary system	Activates cofilin through dephosphorylation	Niwa et al., 2002; Jang et al., 2003
		Required for normal progress through mitosis and normal cytokinesis.			
*Rpl36a*	*60S ribosomal protein L36a*	Structural constituent of ribosome	E2 to E4.5—blastocyst	Down-regulated in spinal cord of Amyotrophic Lateral Sclerosis (ALS) model	de Oliveira et al., 2013; MGI database
			E12.5 to E18.5—brain		
*Tspan2*	*Tetraspanin-2*	Regulation of cell development, activation, growth and motility	E14.5—pituitary	Involved in early differentiation of oligodendrocytes	Birling et al., 1999; Diez-Roux et al., 2011
			Post-natal—CNS		
*Zfp157*	*Zinc finger protein 157*	Stat6-regulated KRAB domain zinc finger protein, binds DNA and regulate transcription	E8.5—head fold, neural tube at mid-gestation—brain, neural tube, mammary placodes, limbs		Oliver et al., 2013
*Snord38a*	*Small nucleolar RNA, C/D Box 38A*	2′O-ribose methylation of RNA	N/A	N/A	Nicoloso et al., 1996

**Figure 2:**
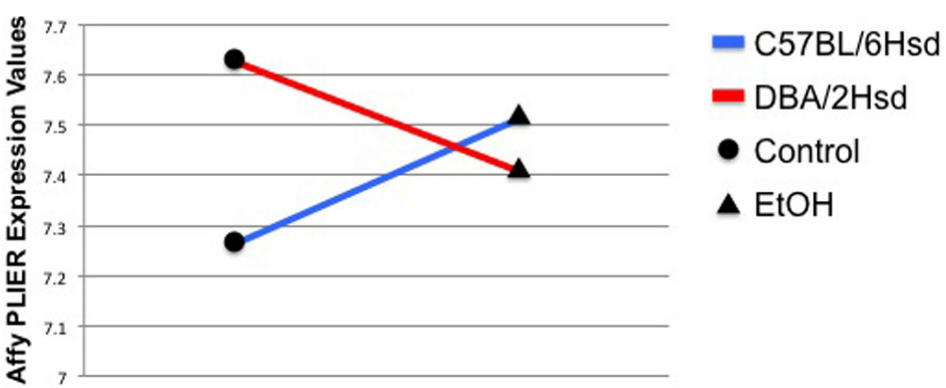
***Eya2* demonstrates a strong GxE interaction**. The Affymetrix PLIER expression levels are shown on the Y-axis. The black circles represent gene expression levels in control embryos, while the triangles represent gene expression levels in the alcohol-treated animals. Two conditions are measured in this experiment, gene expression levels in controls and gene expression levels in EtOH-treated animals. The blue line depicts the changes in gene expression found between the C57BL/6NHsd control embryos (black circle) and their EtOH-treated test embryos (black triangle). The red line depicts the changes in in gene expression found between the DBA/2NHsd control embryos (black circle) and their EtOH-treated test embryos (black triangle). The interaction is depicted by showing an inverse correlation between the gene expression profiles of the two inbred strains.

Genes exhibiting a GxE interaction show not only differential expression, but also opposite patterns of expression that are dependent upon treatment. For example, in NTC embryos, *Eya2* [eyes absent 2 homolog (drosophila)] expression changes from an average of 7.26 ± 0.045 in control C57BL/6NHsd embryos to 7.52 ± 0.004 in ethanol-treated embryos. Conversely, control DBA/2NHsd embryos show an expression value of 7.63 ± 0.007, which drops to 7.40 ± 0.04 following incubation in alcohol (Figure 2). The Affymetrix PLIER expression levels are shown on the Y-axis. The black circles represent gene expression levels in control embryos, while the triangles represent gene expression levels in the alcohol-treated animals. There is a clear inverse correlation between the two genotypes following exposure to alcohol, as expression of *Eya2* increases following treatment in C57BL6NHsd embryos, while it decreases in DBA/2Hsd embryos.

We plotted the GxE interactions for the remaining genes. Twelve of the NTC genes (*Csmd3, Cxadr, D5ert577e, Eno2, Mageb18, Olfr1248, Olfr148, Rhoc, Rpl36a, Tspan2, Vmn1r37, and 7402416P09Rik*) showed strong GxE interactions (Supplemental Data, Figure S1), as measured by an intersecting line demonstrating inverse correlation with gene expression; in a weak interaction, the lines do not intersect, while nine NTC genes (*Fam174b, Marveld2, Mcat, Nudt8, Pdxp, Tac2, Zfp157, 4930579K19Rik, and B230319C09Rik*) showed weak GxE interactions (Supplemental Data, Figure S1). Eleven of the NTO genes (*Apold1, Eya4, Kera, Leo1, Olfr312, Olfr975, Psme4, Qars, Snord38a, Tnfrsf22, and Vps51*) showed strong GxE interactions and two (*Pkm2* and *Vipr1*) showed weak GxE interactions (Supplemental Data, Figure S2).

One concern for gene expression studies using microarrays is whether or not there is inherent expression bias due to SNVs within the probe-sets that hamper binding. This is potentially exacerbated when comparing changes in expression from a strain that is closely related to the reference sequence (i.e., C57BL/6NHsd) to a strain that is more distantly related (i.e., DBA/2NHsd). A hallmark of biased expression would be a consistent observation of decreased signal from the DBA/2NHsd animals in transcripts harboring SNVs in the probe-sets. In Figure 2, the DBA/2NHsd animals show higher levels of *Eya2* expression under control conditions. Therefore, it is highly unlikely that SNVs within the *Eya2* probe-sets are causing biased detection. Examination of the remaining GxE genes shows that 3/35 genes demonstrating GxE interactions potentially exhibit biased expression; *Mcat* and *Zfp157*, which were detected in NTC animals (Figure S1), and *Pkm2*, which was detected in NTO studies (Figure S2), are expressed at lower levels in DBA/2NHsd embryos under both conditions. Examination of the probe-sets for these three genes indicates that there is low likelihood that SNVs within the probe-sets account for the decreased expression in DBA/2NHsd cultures. There are 25 probe-sets that span *Mcat*; 2 of these harbor SNVs. Similarly, 23 probe-sets span *Zfp157*, 2 of which have SNVs. *Pkm2* has 15 probesets, but non-contain any informative SNVs.

### Genomic signatures of the differentially expressed GxE genes

To better understand the genetic basis driving these GxE interactions, we systematically identified the single nucleotide variants (SNVs) between C57BL/6 and DBA/2 using a genomics approach. However, the strains used for the microarray study (C57BL/6NHsd and DBA/2NHsd) were raised at Harlan and have not been added to the mouse sequence database. The closest matches in the mouse database are C57BL/6NJ (Yalcin et al., 2012) and DBA/2J. Therefore, we delineated the SNVs between C57BL/6NJ and DBA/2J in the promoters (5 kb upstream of each transcriptional start site) of all 35 genes showing GxE interactions. We found that 16 of these genes contain SNVs between C57BL/6NJ and DBA/2J: *Eya2, Csmd3, Mcat, Tac2, Vps51, Apold1, Leo1, Psme4, Vipr1, Fam174b, Megab18, Pdxp, Rpl36a, Tspan2, Zfp157, and Snord38a* (Table 2). A number of olfactory receptor genes were also identified, but were placed on the backburner since they display high sequence identity among orthologs, confounding SNV interpretation.

One gene identified in the NTC subtype, *Eya2*, stood out for further examination. *Eya2* exhibits strong GxE interactions and is expressed in the cranial placodes, branchial arches and the CNS during organogenesis (Xu et al., 1997), areas known to be affected clinically in FAS/FASD. There are three alternative transcriptional start sites associated with *Eya2*. To ensure complete analysis, we examined the most proximal 5 kb from all three promoters. The genomic signature profile demonstrates that there are 21 SNVs (seven are in dbSNP Build 138) between C57BL/6NJ and DBA/2NJ within the three promoters (see Table 3). Table 3 provides the genomic location of each SNV, its relative position within the locus and the allele frequency. In addition, Table 3 shows the genomic sequence surrounding each SNV and illustrates changes in putative transcription factor (TF) binding sites and CpG dinucleotides.

**Table 3:** **SNVs near *Eya2*. *Eya2* is on MMU 2 and expressed from the Watson (+) strand**.

	**dbSNP**	**Genomic**	**Start site**			**CpG Site**	**Putative TF binding sites**			**C57BL/6NJ**		**DBA/2J**		**NCBI37/mm9**
**#**	**Build 138**	**Location**	**TSS1**	**TSS2**	**TSS3**	**B6/D2**	**B6**	**D2**	**Flanking sequence**	**NT**	**%**	**NT**	**%**	**NT**
1		165417649	Promoter	Promoter	Promoter		N/A	N/A	GTGTACATGG[T/C]ATATGTATGG	T	100	C	100	T
2	rs33341731	165419967	Promoter	Promoter	Promoter	CG/CA	Hes1	Hoxa5/Hes1	GAAACCTTGC[G/A]TTGGGGGGTG	G	100	A	100	G
3		165429834	Intron 1	Promoter	Promoter		SRF	N/A	TATAACCCGA[G/A]CTTAAAGGCC	G	100	A	100	G
4	rs33448850	165431347	Intron 1	Promoter	Promoter	CG/TG	N/A	TFE3-S/c-Fos	TATACTCACA[C/T]GAAAAAAAAC	C	100	T	100	C
5		165465686	Intron 1	Promoter	Promoter		C/EBPbeta	c-Fos	ACACACACAC[A/T]CACACACACA	A	100	T	100	A
6		165466018	Intron 1	Promoter	Promoter		C/EBPbeta c-Fos	TCF-1(P) c-Fos	TCTCTCTCTC[A/T]CACACACACA	A	100	T	100	A
7		165475306	Intron 1	Promoter	Promoter		N/A	C/EBPbeta	GGGATGGAGG[G/A]CAGATAAGAA	G	100	A	100	G
8		165475312	Intron 1	Promoter	Promoter		GATA-1	Tal-1 RelA	GAGGGCAGAT[AA/GG]GAAGGGACA	A	100	G	100	A
							GATA-2	NF-kappaB COE1						
9		165475313	Intron 1	Promoter	Promoter					A	100	T	100	A
10		165475318	Intron 1	Promoter	Promoter		N/A	TCF-1(P);	AGATAAGAAG[G/A]GACAGGGAGA	G	100	A	100	G
11		165475328	Intron 1	Promoter	Promoter		TCF-1(P)	c-Fos DEC2	GGACAGGGAG[A/G]TGAATGGGAT	A	100	G	100	A
12	rs27298255	165485303	Intron 1	Intron 1	Promoter		N/A	HES-1 myogenin	GTGTTAACAG[G/C]AAGTTGTGAA	G	100	C	100	G
								MyoD						
13		165485789	Intron 1	Intron 1	Promoter		N/A	N/A	CCACCACCAC[T/C]ATACAAAGGA	T	100	C	100	T
14		165486306	Intron 1	Intron 1	Promoter		TCF-1(P)	C/EBPbeta	CTCTCTCTCT[C/G]TGTGTGTGTG	C	100	G	100	C
								TCF-1(P)						
15		165488467	Intron 1	Intron 1	Promoter		N/A	c-Fos DEC2	AGATGGGTTG[G/A]AGCTAGCCTG	G	100	A	100	G
16		165488503	Intron 1	Intron 1	Promoter	GG/CG	LyF-1	LyF-1	GTCGTTGTTT[G/C]GGGAGAAATC	G	100	C	100	G
17	rs3718350	165488796	Intron 1	Intron 1	Promoter		GR	TCF-1(P)	CTTAACAAGA[A/G]CAGTATGCAC	A	100	G	100	A
							C/EBPbeta							
18	rs27298227	165489658	Intron 1	Intron 1	Promoter		TFE3-S	TFE3-S	GTCTCACATG[GG/CA]TAAGGCAAGA	G	100	C	100	G
19		165489659	Intron 1	Intron 1	Promoter					G	100	A	100	G
20	rs27298226	165489698	Intron 1	Intron 1	Promoter	CG/CA	TCF-1(P)	c-Fos	TAGCCCTCTC[G/A]GTACTTAGCA	G	100	A	100	G
								TCF-1(P)						
21	rs27298225	165489830	Intron 1	Intron 1	Promoter	CG/AG	HES-1	HES-1	AAGGCACTTG[C/A]GAAACAGTAT	C	100	A	100	C
							Nkx2-1	Nkx2-1 c-Fos						

Sixteen of the twenty-one SNVs identified in the three promoters of *Eya2* potentially lead to the loss or gain of putative transcription factor (TF) binding sites. For example, there are no predicted TF binding sites at SNVs 1, 4, 7, 10, 13, 14, and 16 in the C57BL/6NJ allele, while SNVs 4, 7, 10, 13, and 16 are predicted to contain one or more TF binding sites in the DBA/2J allele. Given the potential for these SNVs to function not only in a genetic manner, but also epigenetically through alterations in DNA methylation, we report that 5 of the 21 SNVs within the three promoters of *Eya2* change a CpG dinucleotide. Four of these (SNV 2, 4, 21, and 22) lead to the loss of a CpG dinucleotide in the DBA/2J background. Examination of putative TF binding sites in these four SNVs shows that there are fewer potential TF binding sites in the C57BL/6NJ allele compared to the DBA/2J allele. *Eya2* shows increased expression in C57BL/6NHsd embryos following alcohol treatment, while the DBA/2NHsd animals exhibit a decrease in expression following alcohol exposure. This would be consistent with increased TF binding in C57BL/6NHsd embryos compared to DBA/2NHsd cultures, and provides a putative mechanism for the GxE interactions.

Similar SNV analyses of the other 15 genes are documented in the Supplemental Files (Table S1). There are four genes that show strong GxE interactions that also have SNVs within the proximal promoter: *Apold1* (Apolipoprotein L domain containing 1), *Leo1* (Paf1/RNA Polymerase II Complex Component*), Psme4* (Proteasome Activator Subunit 4) and *Snord38a* (Small nucleolar RNA, C/D box 38A) (Table 2). *Apold1* is thought to be involved in angiogenesis in the brain vasculature (Diez-Roux et al., 2011; Zhou et al., 2013); *Leo1*, which important in chromatin remodeling, is involved in neural crest development (Wertz and Herrmann, 2000); *Psme4* is a component of the proteasome that specifically targets histones for degradation following DNA damage (Ustrell et al., 2002; Qian et al., 2013); and *Snord38a* is a C/D box small nucleolar RNA that is important for 2′O-ribose methylation of rRNAs (Nicoloso et al., 1996).

A summary of the putative regulatory implications of these 241 SNVs is found in Table 4. Ten genes were found in the NTC subtype, while six were detected in the NTO embryos. Out of the 241 SNVs identified between C57BL/6NJ and DBA/2J, 186 have the possibility to alter TF binding sites, while 62 cause the addition or removal of a CpG dinucleotide. Out of the 62 SNVs that lead to CpG changes, 34 (55%) led to the loss of a CpG dinucleotide, 26 (42%) led to the *de novo* creation of a CpG site and 2 (3%) changed the position of a CpG site (i.e., CCG to CGG). In addition, the vast majority of SNVs that alter a CpG site [53/62 (85%)] are associated with potential additions, losses and/or substitutions in TF binding sites, indicating that both genetic (SNVs) and epigenetic (CpG methylation) could be implicated in the GxE interactions.

**Table 4:** **Summary of the potential regulatory effects of the SNVs between C57BL/6NJ and DBA/2J in genes demonstrating GxE interactions**.

**NTC/NTO**	**Symbol**	**Gene**	**MMU**	**Coordinates**	**Strand**	**B6/D2 SNV**	**Altered TFB**	**Potential epigenetic gene regulation changes**			
								**CpG Change**	**CG to NN**	**NN to CG**	**Altered TFB**
NTC	*Csmd3*	CUB and Sushi multiple domains 3	15	47412184-48623535	Reverse	11	8	1	0	1	1
NTC	*Eya2*	Eyes Absent	2	165420528-165597131	Forward	21	16	5	4	1	4
		Homolog2 (Drosophila)									
NTC	*Fam174b*	Membrane protein	7	80885193-80921805	Forward	1	0	0	0	0	0
		FAM174B precursor									
NTC	*Mageb18*	Melanoma-associated antigen	X	89364218-89844911	Forward	1	1	0	0	0	0
		B18									
NTC	*Mcat*	Malonyl CoA:ACP	15	83377227-83386141	Reverse	54	42	12	6	5	9
		Acyltransferase									
		(Mitochondrial)									
NTC	*Pdxp*	Pyridoxal phosphate phosphatase	15	78744349-78749947	Forward	18	14	9	5	4	8
NTC	*Rpl36a*	60S ribosomal protein L36a	X	131120193-131122601	Forward	3	2	1	1	0	1
NTC	*Tac2*	Tachykinin-2	10	127162448-127168824	Forward	9	7	3	1	2	3
NTC	*Tspan2*	Tetraspanin-2	3	102538693-102576233	Forward	1	1	0	0	0	0
NTC	*Zfp157*	Zinc finger protein 157	5	138882704-138901922	Forward	5	3	2	2	0	1
NTO	*Apold1*	Apolipoprotein L	6	134932019-134936854	Forward	51	43	12	6	6	11
		Domain Containing 1									
NTO	*Leo1*	Paf1/RNA	9	75289331-75314239	Forward	6	6	3	1	2	3
		Polymerase II									
		Complex Component									
NTO	*Psme4*	Proteasome (Prosome, Macropain) Activator	11	30671775-30780361	Forward	16	10	2	1	1	2
		Subunit 4									
NTO	*Snord38a*	Small nucleolar RNA, C/D box 38A	4	116827121-116827179	Reverse	32	24	8	4	3	7
NTO	*Vipr1*	Vasoactive Intestinal	9	121551834-121582072	Forward	8	6	3	2	1	2
		Peptide Receptor 1									
NTO	*Vps51*	Vacuolar protein sorting 51	19	6067842-6077187	Reverse	4	3	1	1	0	1
Total						241	186	62	34	26	53

To better understand the genetic variability between the C57BL/6 sub-strains used for gene expression studies (C57BL/6NHsd) and SNV analysis (C57BL/6NJ), we used the SNV Data from the Broad Institute (Broad2) to identify SNVs between C57BL/6NHsd and C57BL/6NJ in the “Compare Two or More Strains” option from the Mouse Phenome Database (http://phenome.jax.org/db/q?rtn=strains/search&compare2=1). We discovered that there are 119590 SNVs between C57BL/6NHsd and C57BL/6NJ, which given a genome of ~ 2.7 × 10^9^ bp, corresponds to a mutation frequency of 4.4 × 10^−5^ mutations/bp, or one SNV in every ~22,500 bp. In this study, we analyzed 5000 bp from 18 promoters of 16 genes, including three promoters for *Eya2*. This totals 90,000 bp of sequence. Given a mutation frequency of 4.4 × 10^−5^, we would expect to find four SNVs that could be attributed to background alterations between C57BL/6NHsd and C57BL/6NJ in our sample. Although we need to estimate the background mutation frequency between DBA/2NHsd (expression studies) and DBA/2NJ (SNV analysis), the DBA/2NHsd line has not been sequenced nor has it been included in any of the available SNP chip repositories. Therefore, we assumed a similar mutation frequency between the two DBA/2 lines (i.e., four mutations in 90,000 bp). Taking these numbers as an estimate, we would expect to find eight total SNVs (four for the C57BL/6 lines and four for the DBA/2 lines) that could be due within sub-line mutations. However, we found 241 SNVs between C57BL/6NJ and DBA/2J in this study, which corresponds to a mutation frequency of 2.7 × 10^−3^ mutations/bp, indicating that likelihood of seeing a GxE interaction due to false positives (~0.033) falls within a 0.05 confidence interval.

## Discussion

This study was conducted to better understand the genetic underpinnings underlying the susceptibility to alcohol exposure during fetal development. We pursued this by identifying the intersected set of genes that: (1) Are differentially expressed in response to alcohol treatment during neurulation; and (2) Demonstrate a genetic interaction between two strains of mice (C57BL/6 and DBA/2) that respond very differently to prenatal alcohol exposure. While C57BL/6 animals are highly sensitive to the teratogenic effects of alcohol, DBA/2 animals are remarkably resistant, as shown by studies using gavage feeding (Gilliam et al., 1988; Downing et al., 2009, 2012) and our prior work in cultured embryos that found alcohol-vulnerability in C57BL/6 embryo cultures exposed to a short alcohol incubation period (Ogawa et al., 2005; Chen et al., 2011; Zhou et al., 2011).

These comparative expression studies reveal a cohort of ~1400 genes that are preferentially expressed in either the C57BL/6NHsd or DBA/2NHsd strains during the beginning stages of neurulation. These genes may represent a first tier contribution to either protect (DBA/2NHsd) or increase (C57BL/6NHsd) the vulnerability of the embryo to the effects of alcohol exposure. Because this set of transcripts does not take alcohol exposure into consideration (e.g., C57BL/6NHsd ± EtOH vs. DBA/2NHsd ± EtOH), it represents the cohort of transcripts that differ between these two strains in two independent experiments. Genes of particular interest are the neural transcription factor and homeobox genes (*Nkx1-2, Sox21, Igf1*, and *Gbx2*), as they can mediate neural progenitor cell fate determination and neural tube patterning. The potassium channel (*Kcnmb1*) and cholinergic receptor (*Chnb1*) genes can mediate timely signal transduction during neural differentiation, while *Hist1h2ab* and *Setd7* have the potential to differentially affect the epigenetic response of the developing brain cells to environmental assaults, including alcohol. The apoptosis, cell cycle and heat shock proteins act to influence cell number, and could contribute to neural tube deficits between the two lines.

For stringent comparison, the closed neural tube (NTC) and open neural tube (NTO) embryonic subtypes were analyzed independently, with an FDR of 5% as the cut off. These differentially expressed genes potentially network to alter inflammatory response, cell death and survival, energy production, embryonic development and hematopoiesis between the two inbred lines. This cohort of differentially expressed transcripts likely results from combinations of factors including genetic background, innate programming, reactivation of transposable elements and epigenetic inheritance and responses. Since this study was conducted on embryonic cultures, it eliminates maternal elements (e.g., physiology, maternal care, circulation factors) that would have normally acted during this critical developmental window. However, since we analyzed whole brains, we were not able to assess tissue-specificity or cell-specific differences in gene expression, and it is plausible that we missed rare transcripts with large effects or primary cell-specific transcripts that are driving the genetic differences in the sensitivity of these two inbred strains to alcohol exposure. Therefore, it is likely that our results are an under-representation of the total number of genes that are differentially expressed between these two lines.

Genes exhibiting GxE interactions demonstrate differential expression that inversely correlates with genotype and exposure to the environmental factor (i.e., alcohol). With an FDR of 5%, we identified 35 genes that exhibited GxE interactions, as measured by an inverse correlation in gene expression (based on genotype) in response to alcohol. Examination of potential mechanisms for GxE interactions indicates that 16 genes have SNVs in their proximal promoters. Several of these 16 candidates are strongly associated with brain and cranial development, including preplacedal ectoderm differentiation (*Eya2*) (Xu et al., 1997; Grifone et al., 2007; Sato et al., 2010), cardiac and neural crest development (*Leo1*) (Wertz and Herrmann, 2000), neuronal survival (*Vipr1*) (Delgado et al., 1996; Fabricius et al., 2011) and oligodendryocyte differentiation (*Tspan2*) (Birling et al., 1999; Diez-Roux et al., 2011). Others are implicated in behavioral disorders including autism spectrum disorders (*Csmd3* and *Fam147b*) (Shimizu et al., 2003; Floris et al., 2008; Diez-Roux et al., 2011; Sarahan et al., 2011; Kamien et al., 2014), Amytrophic Lateral Sclerosis (*Rps36a*) (de Oliveira et al., 2013) and pain modulation (*Tac2*) (Mar et al., 2012). Three genes in the NTO subtype are involved in gene regulation, *Leo1* (chromatin structure and gene regulation; Wertz and Herrmann, 2000), *Psme4* (proteasome degradation of histones; Ustrell et al., 2002; Qian et al., 2013) and *Snord38a* (modifications of ribosomal RNA; Nicoloso et al., 1996). Although it is not clear if there are any specific pathways that are affected by these four genes, several have the ability to disrupt expression and protein function on a global scale. Most of these are downregulated in C57BL/6NHsd animals following alcohol exposure, indicating that global disruption to the proteasome pathway, chromatin remodeling and rRNA methylation may be a factor in the increased developmental delay in the NTO embryonic cultures.

It is becoming increasingly clear that phenotypic variability and causative lesions are not restricted to the protein-coding regions of the genome. Several GWAS studies have demonstrated that SNVs in non-coding regions of the DNA are strongly associated with disease processes (De Gobbi et al., 2006; Enjuanes et al., 2006; Choi et al., 2009; Glinskii et al., 2009; Heckmann et al., 2010; Maceachern et al., 2011a,b; Brown et al., 2013; Fan et al., 2013; Perumbakkam et al., 2013; Renteria et al., 2013).

To understand the possible regulatory consequences associated with these gene expression changes, we took this analysis one step further by cataloging the SNVs between the parental strains in the promoter regions of the 35 genes and determining potential regulatory changes associated with the SNVs. Sixteen of these genes had a least one SNV in the proximal promoter (i.e., within 5 kb of the transcriptional start site), for a total of 241 SNVs within 90,000 bp of promoter sequence. The majority of SNVs (77%; 186/241) altered the genomic DNA in a way that led to the predicted addition or subtraction or a potential transcription factor binding site, indicating that these underlying genetic differences could play significant roles in the differential phenotypes we detected in this study. In addition, ~25% (62/241) of the SNVs have the potential to affect DNA methylation by either creating or eliminating a CpG dinucleotide. Since DNA methylation is affected by alcohol exposure *in utero* (Ramsay, 2010; Laufer et al., 2013; Resendiz et al., 2013; Ungerer et al., 2013) and epigenetic gene regulation is critical for neuronal development (Chen et al., 2014), the underlying genetic differences between the C57BL/6 and DBA/2 inbred strains that impact CpG dinucleotides could create epigenetic consequences that predispose the C57BL/6 embryos to the teratogenic effects of alcohol, while protecting DBA/2 from this vulnerability.

## Conclusions

By using a multi-pronged approach in two separate experiments (neural tube open and neural tube closed phenotypes), we captured over 900 genes that have the potential to contribute to the cascade that leads to differential dysmorphology in embyros from the two genetically contrasting lines. These first tier genes are good candidates for further understanding the phenotypic variability associated with *in utero* alcohol exposure. Exploration of the genetic and environmental factors led us to a cohort of 35 genes subject to GxE interactions; these genes represent the best candidates for driving the downstream morphological changes observed following alcohol exposure. Examination of potential regulatory SNVs associated with these 35 candidates indicates that the differences between the C57BL/6 and DBA/2 inbred strains could be due to a variety of factors, including binding of *cis*-acting elements (e.g., transcription factors), expression of *trans*-acting factors, epigenetic events, evolutionary consequences and combinations of these different factors.

These experiments lay the groundwork for future studies aimed at testing the causality of these different SNVs. Although a seemingly daunting task, with emerging genome modification tools, such as zinc finger, TALEN and CRISPR modification systems (Gaj et al., 2013) under development and refinement, it may be possible to start systematically disrupting multiple SNVs with exquisite sensitivity. Other possible approaches include using selection to screen for animals containing the most predictive SNVs and determining which cohorts of SNVs recapitulate the susceptible phenotype in the resistant DBA/2NHsd population. Studies in chicken demonstrate the feasibility and elegance of this approach (Maceachern et al., 2011a,b; Perumbakkam et al., 2013). Although we cannot rule out that the alterations in gene expression are not due to the SNVs identified here, this study provides the top 35 genes showing GxE interactions following alcohol exposure, providing a strong platform for future studies aimed at understanding the roles of these genetic and transcriptional changes associated with vulnerability to the teratogenic effects of fetal alcohol exposure.

### Conflict of interest statement

The Guest Associate Editor Stephen Bruce Mason declares that, despite being affiliated to the same Institution as authors Feng C. Zhou and Chiao-Ling Lo, the review process was handled objectively. The authors declare that the research was conducted in the absence of any commercial or financial relationships that could be construed as a potential conflict of interest.

## Supplementary material

Figure S1 **Gene x Environment interactions of the genes identified in the embryos with closed neural tubes (NTC)**. The Affymetrix PLIER expression levels are shown on the Y-axis. The black circles represent gene expression levels in control embryos, while the triangles represent gene expression levels in the alcohol-treated animals. Two conditions are measured in this experiment, gene expression levels in controls and gene expression levels in EtOH-treated animals. The blue line depicts the changes in gene expression found between the C57BL/6NHsd control embryos (black circle) and their EtOH-treated test embryos (black triangle). The red line depicts the changes in in gene expression found between the DBA/2NHsd control embryos (black circle) and their EtOH-treated test embryos (black triangle). The interaction is depicted by showing an inverse correlation between the gene expression profiles of the two inbred strains.

Figure S2 **Gene x Environment interactions of the genes identified in the embryos with open neural tubes (NTO)**. The Affymetrix PLIER expression levels are shown on the Y-axis. The black circles represent gene expression levels in control embryos, while the triangles represent gene expression levels in the alcohol-treated animals. Two conditions are measured in this experiment, gene expression levels in controls and gene expression levels in EtOH-treated animals. The blue line depicts the changes in gene expression found between the C57BL/6NHsd control embryos (black circle) and their EtOH-treated test embryos (black triangle). The red line depicts the changes in in gene expression found between the DBA/2NHsd control embryos (black circle) and their EtOH-treated test embryos (black triangle). The interaction is depicted by showing an inverse correlation between the gene expression profiles of the two inbred strains.
