# Supplementary figures and images for: Implications of genomic signatures in the differential vulnerability to fetal alcohol exposure in C57BL/6 and DBA/2 mice
Source: Front Genet. 2014 Jun 11;5:173. doi: 10.3389/fgene.2014.00173 (PMC4052096; doi:10.3389/fgene.2014.00173)

# Supplemental Figure 1

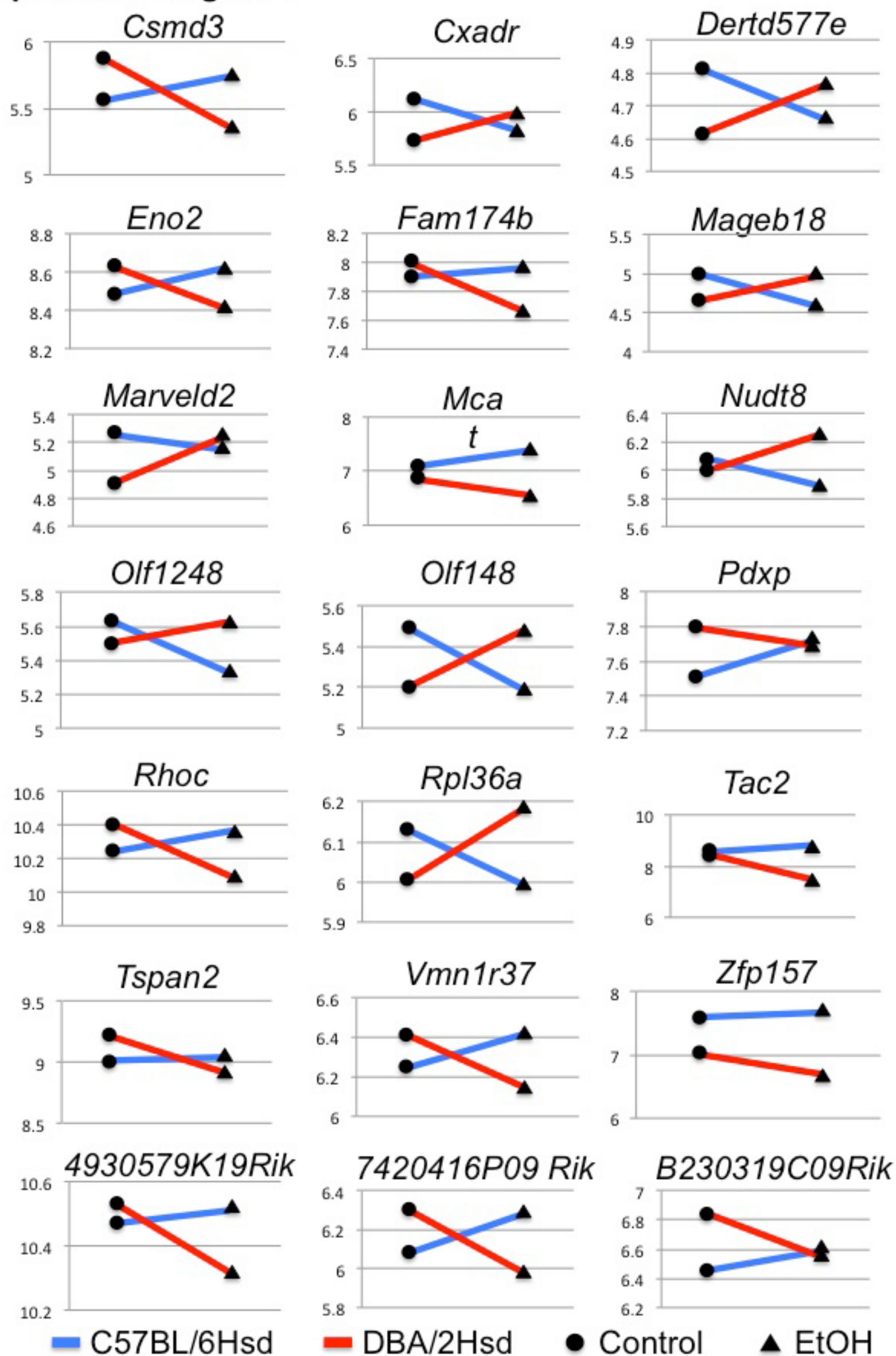

Supplement: Figure S1 — Gene x Environment interactions of the genes identified in the embryos with closed neural tubes (NTC). The Affymetrix PLIER expression levels are shown on the Y-axis. The black circles represent gene expression levels in control embryos, while the triangles represent gene expression levels in the alcohol-treated animals. Two conditions are measured in this experiment, gene expression levels in controls and gene expression levels in EtOH-treated animals. The blue line depicts the changes in gene expression found between the C57BL/6NHsd control embryos (black circle) and their EtOH-treated test embryos (black triangle). The red line depicts the changes in in gene expression found between the DBA/2NHsd control embryos (black circle) and their EtOH-treated test embryos (black triangle). The interaction is depicted by showing an inverse correlation between the gene expression profiles of the two inbred strains. [file Presentation1.PDF]

*Apold1*

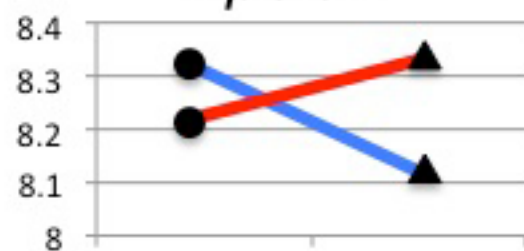

*Eya4*

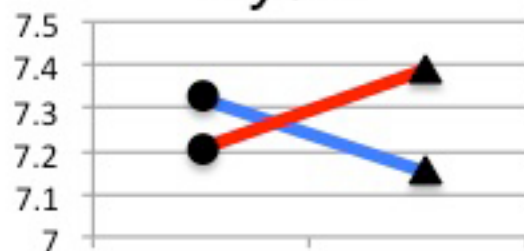

*Kera*

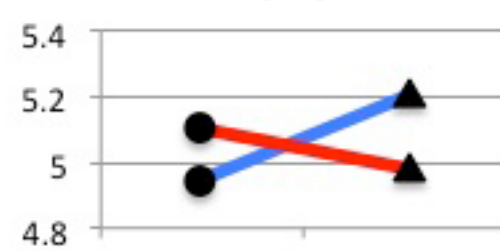

*Leo1*

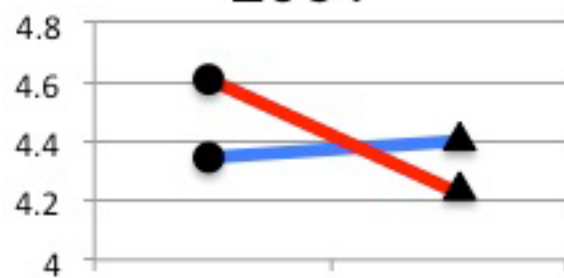

*Olf312*

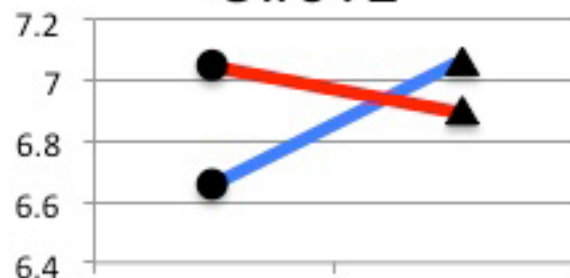

*Olfr975*

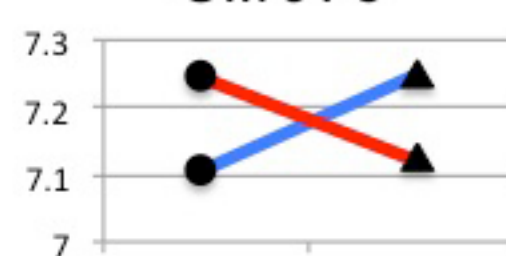

*Pkm2*

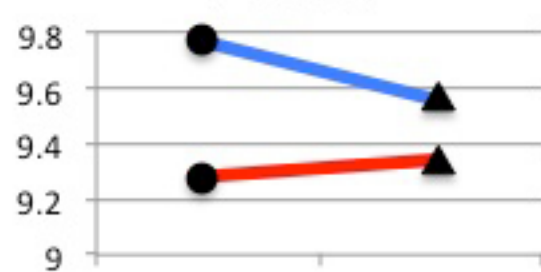

*Psme4*

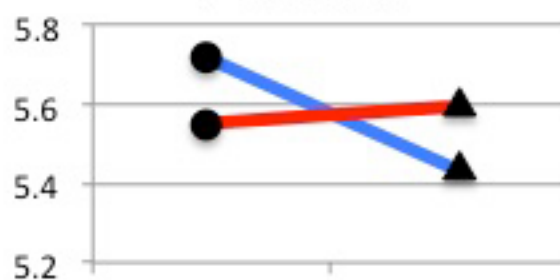

*Qars*

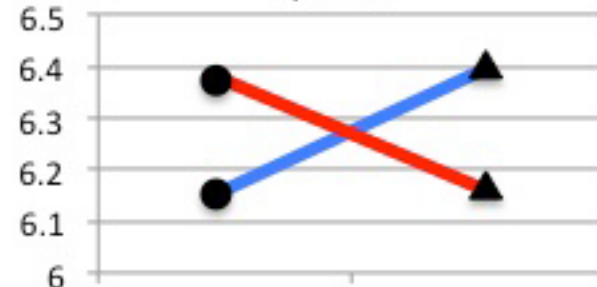

*Snord38*

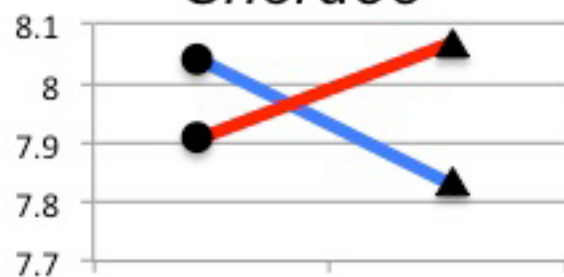

*Tnfrsf22*

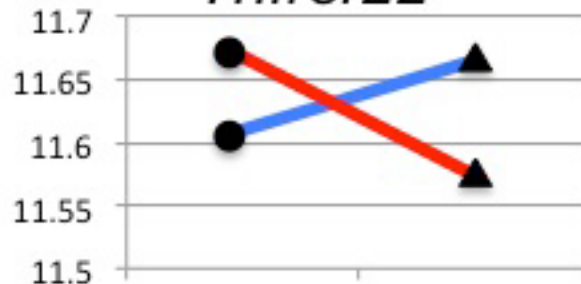

*Vipr1*

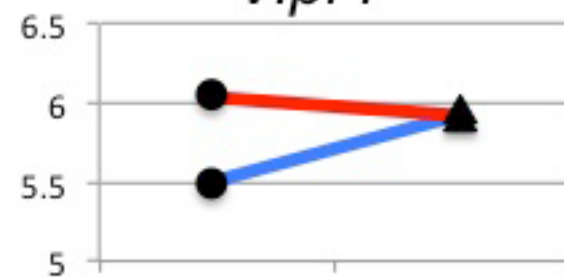

*Vps51*

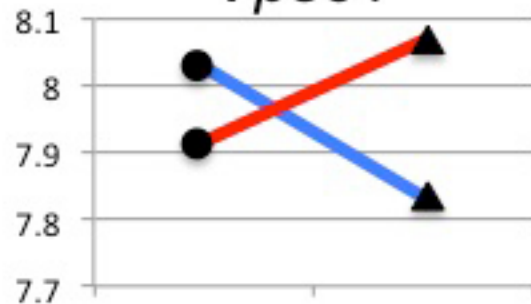

— C57BL/6Hsd

— DBA/2Hsd

● Control

▲ EtOH

Supplement: Figure S2 — Gene x Environment interactions of the genes identified in the embryos with open neural tubes (NTO). The Affymetrix PLIER expression levels are shown on the Y-axis. The black circles represent gene expression levels in control embryos, while the triangles represent gene expression levels in the alcohol-treated animals. Two conditions are measured in this experiment, gene expression levels in controls and gene expression levels in EtOH-treated animals. The blue line depicts the changes in gene expression found between the C57BL/6NHsd control embryos (black circle) and their EtOH-treated test embryos (black triangle). The red line depicts the changes in in gene expression found between the DBA/2NHsd control embryos (black circle) and their EtOH-treated test embryos (black triangle). The interaction is depicted by showing an inverse correlation between the gene expression profiles of the two inbred strains. [file Presentation2.PDF]
